# Genome Analysis of ESBL-Producing *Escherichia coli* Isolated from Pigs

**DOI:** 10.3390/pathogens11070776

**Published:** 2022-07-07

**Authors:** Luria Leslie Founou, Raspail Carrel Founou, Mushal Allam, Arshad Ismail, Sabiha Yusuf Essack

**Affiliations:** 1 Antimicrobial Research Unit, University of KwaZulu-Natal, Durban 4000, South Africa; czangue@yahoo.fr (R.C.F.); essacks@ukzn.ac.za (S.Y.E.); 2 Reproductive, Maternal, Newborn and Child Health (ReMarch) Research Unit, Research Institute of the Centre of Expertise and Biological Diagnostic of Cameroon (CEDBCAM-RI), Yaoundé P.O. Box 8242, Cameroon; 3 Bioinformatics and Applied Machine Learning Research Unit, EDEN Foundation, Yaoundé P.O. Box 8242, Cameroon; 4 Department of Microbiology, Haematology and Immunology, Faculty of Medicine and Pharmaceutical Sciences, University of Dschang, Dschang P.O. Box 96, Cameroon; 5 Antimicrobial Resistance and Infectious Diseases (ARID) Research Unit, Research Institute of the Centre of Expertise and Biological Diagnostic of Cameroon (CEDBCAM-RI), Yaoundé P.O. Box 8242, Cameroon; 6 Sequencing Core Facility, National Health Laboratory Service, Johannesburg 2131, South Africa; mushalallam@gmail.com (M.A.); arshardi@nicd.ac.za (A.I.); 7 Department of Genetics and Genomics, College of Medicine and Genomics, Al Ain 15551, United Arab Emirates

**Keywords:** Antibiotic resistance, ESBL-*E. coli*, genomics, food safety

## Abstract

The resistome, virulome and mobilome of extended spectrum ß-lactamase (ESBL)-producing *Escherichia coli* (ESBL-Ec) isolated from pigs in Cameroon and South Africa were assessed using whole genome sequencing (WGS). Eleven clonally related phenotypic ESBL-*Ec* isolates were subjected to WGS. The prediction of antibiotic resistance genes, virulence factors (VFs) and plasmids was performed using ResFinder, VirulenceFinder and PlasmidFinder, respectively. Diverse sequence types (STs) were detected with ST2144 and ST88 being predominant and *bla_CTX-M-15_* (55%) being the principal ESBL gene. All except two isolates harboured various aminoglycoside resistance genes, including *aph(3″)-Ib* (6/11, 55%) and *aph(6)-1d* (6/11, 55%), while the *qnrS1* gene was identified in four of the isolates. The ESBL-*Ec* isolates showed a 93.6% score of being human pathogens. The *fim*, *ehaB*, *ibeB/C* were the leading virulence factors detected. All isolates harboured at least three extraintestinal pathogenic *E. coli* (ExPEC) VFs, with one isolate harbouring up to 18 ExPEC VFs. Five isolates (45.45%) harboured the plasmid incompatibility group IncF (FII, FIB, FIC, FIA). The study revealed that there is an urgent need to implement effective strategies to contain the dissemination of resistant and virulent ESBL-*Ec* through the food chain in Cameroon and South Africa.

## 1. Introduction

Antibiotic resistance (ABR) is a global public health issue that has severe multi-dimensional repercussions not only in humans, but also in the food production industry. The extensive use of antibiotics in food animal production is widely acknowledged as the driving force behind antibiotic resistance in humans and animals. Antibiotics are used for a variety of purposes, including therapeutic and non-therapeutic uses, as metaphylactics, prophylactics, and growth promoters [1]. Hence, the emergence and spread of ABR across the farm-to-plate continuum puts occupationally exposed workers (viz. farmers, agricultural practitioners, abattoir workers, food handlers, etc.), their close contacts and consumers at the end of the food chain at risk of contamination or infection by antibiotic-resistant bacteria (ARB) and/or antibiotic resistance genes (ARGs) [1,2]. ABR prevention and containment measures should focus not only on humans but also on animals and their associated environments [2].

*Escherichia coli* is a recognized commensal bacterium of the gastrointestinal tract of humans and animals. The genomic plasticity of *E. coli* strains allows their adaptation to different environments, hence their wide implication in intestinal and extraintestinal infections in both humans and animals worldwide [3]. *E. coli* displays a clonal population structure delineating four main phylogenetic groups (A, B1, B2, and D) with few phylogroups being involved in infections and others being commensal [4,5]. 

*E. coli* has been suggested as the putative reservoir for extended-spectrum β-lactamase (ESBL) resistance. It has been demonstrated that substantial resistance emerges in commensal bacteria, especially those present in the gastrointestinal tract where horizontal gene transfer prevails and does occur within and between species and genera [2]. ESBL production in *E. coli* is associated with different resistance genes but is most frequently caused by the production of ESBLs encoded by the *bla*_TEM_, *bla*_SHV_ and *bla*_CTX-M_ families, with the latter being the predominant type [6]. ESBL-producing *E. coli* (ESBL-*Ec*) have been detected across the animal, human and environmental interface worldwide, with the emergence of specific clones able to acquire ARGs and virulence factors (VFs) via mobile genetic elements (MGEs), such as plasmids, transposons, gene cassettes and other integrative genetic elements [2].

ESBL-*Ec* emerging on farms and/or abattoirs can disseminate directly to occupationally exposed workers, and indirectly through the food chain via contact with or consumption of contaminated food products. This direct and indirect transmission of ESBL-*Ec* facilitate the likelihood of their subsequent entrance and spread into communities and hospitals [7]. This is further exacerbated by international travels and the globalization of trade in animals and food products, meaning that there are no species, nor geographical frontiers to contain such resistant pathogens [7]. Chishimba et al., (2016) and Aworh et al., (2020) reported 20 and 32% of ESBL-*Ec* in poultry abattoirs in Zambia and Nigeria, respectively [8,9]. Both studies concluded that the presence of ESBL-*Ec* in food animals and food products poses a significant public health threat for the general population that requires urgent and appropriate containment measures. 

Understanding the evolution, transmission dynamics of ESBL-*Ec* are thus essential and whole genome sequencing (WGS) has been recognized as a highly discriminatory bacterial typing technique that surpasses previous methods [10]. WGS was for instance used to evidence high resolution and the transmission of ESBL-*Ec* between broilers, farmers and household members in a Dutch farm [11]. 

Despite the evidence of increasing prevalence and the potential zoonotic transmission of ESBL-*Ec* in food animals, there is still limited information regarding the genetic structure, diversity and relationship of ESBL-*Ec* isolates from food animals, especially in the pig industry in sub-Saharan African countries, such as Cameroon and South Africa. 

As part of a previous multi-centre study conducted from March to October 2016 in Cameroon and South Africa, a total of 432 nasal and rectal swabs were collected from pigs in both countries [12]. Three individual swabs were pooled according to the gender, area of breeding, farm and age of animals, leading to 288 pool samples (144 nasal and 144 rectal pools). Upon identification and antimicrobial susceptibility testing, all putative ESBL-*Ec* underwent enterobacterial-repetitive-polymerase chain reaction (ERIC-PCR) where they were grouped into 14 clusters. The ERIC genotypes revealed that isolates spread within and between abattoirs, as well as within and across countries, with some isolates originating from Cameroon being highly related to those from South Africa. In order to assess further the transmission of ESBL-*Ec* across the food chain, within each generated cluster, ESBL-*Ec* isolates having high genetic relationships with those from another abattoir or country were considered for WGS.

The objectives of this study were thus to use WGS and bioinformatics tools to investigate the pathogenicity, genetic diversity and resistome, virulome and mobilome of ESBL-*Ec* isolates from pigs in Cameroon and South Africa in order to ascertain their potential threat in human health.

## 2. Results

### 2.1. Baseline Characteristics and Phenotypic Analyses

All the *E. coli* isolates were ESBL producers and had a high level of resistance to ampicillin, cefuroxime, cefuroxime acetyl, as well as to third (cefotaxime, ceftazidime) and fourth generation (cefepime) cephalosporins were observed (Table S1). All isolates were resistant to trimethoprim–sulfamethoxazole and susceptible to cefoxitin, ertapenem, meropenem, imipenem and tigecycline (Table S1). Two isolates displayed multidrug resistance (MDR; resistance to three or more antibiotic families) with one isolate, PN256E8, being resistant to colistin with a minimum inhibitory concentration (MIC) of 8 mg/L. Relevant population data, specimen source, phenotypic and genotypic characteristics for these isolates are summarized in Table 1. 

### 2.2. Genomic Features

Table 1 and Table S2 depict all the genomic characteristics, including length, GC content, N50, coverage, coding sequences, RNAs, rMLST, phylotype and serotype, of the isolates. The genome size of the isolates ranged from 4.5 Mb to 5.3 Mb with a GC content of 50.5 to 50.9 and coverage of 111 to 188 (Table S2).

### 2.3. Antimicrobial Resistance Phenotypes and Genotypes

Whole genome-based resistome analyses revealed that all isolates evidenced relatively similar combinations of resistance genes encoding target modification, antibiotic inactivation, antibiotic efflux pumps and regulators. Six (54.54%) *E. coli* isolates harboured *bla_CTX-M-15_* with other *bla_CTX-M_*_-group_ being detected in the others. Three isolates simultaneously harboured the *bla_CTX-M-15_* and *bla_TEM-1B_* whilst one isolate, PN256E8 harboured concomitantly the *bla_CTX-M-15_*, *bla_TEM-1B_*, *bla_TEM-141_* and *bla_TEM-206_* (Table 2). 

All except two isolates (PR256E1, PN256E2) harboured various aminoglycoside resistance genes, including *aph(3″)-Ib* (6/11, 55%) and *aph(6)-1d* (6/11, 55%) genes (Table 2). Eight (73%) isolates harboured different *aad* genes, including *aadA5* (3/11; 27%) and *aadA1* (3/11; 27%) (Table 2). Several types of plasmid-mediated quinolone resistance (PMQR) genes were also identified in the isolates (Table 2) with the *qnrS1* gene being detected in four (36%) isolates, whilst the *aac(6′)Ib-cr* and *oqxAB* genes were identified in three isolates (27%) each. Mutations in the *gyrA* quinolone resistance-determining region (*QRDR*) genes were observed in two isolates (PR010E3I, PN091E1II) where three mutations were observed in *gyrA* with two (S**83L**, D**87**N) occurring within PR010E3I and one (S**83**A) in PN091E1II (Table 2). All isolates, except for PN256E8 and PR010E3I, for which the PMQR gene was identified in both and additionally QRDR in the latter, were susceptible to ciprofloxacin.

All isolates displayed concomitant resistance to trimethoprim and sulfamethoxazole with all harbouring at least one *sul* gene variant. Specifically, *sul2* gene was identified in nine (82%) isolates alone and in combination with *sul1* gene in one (PR010E3I). Similarly, the *dfr* gene was identified in 7/11 (64%) isolates, specifically *dfrA17* (n = 3) and *dfrA14* (n = 3). Diverse permutations of *dfr* and *sul* genes occurred in the isolates with *sul2* and *dfrA14* being detected in three (27%) isolates while *sul2* and *dfrA17* were identified in two isolates (Table 2). One isolate (9%) harboured the *mcr-1* gene encoding for colistin resistance (Table 2).

### 2.4. Whole-Genome Virulome Profiling and Pathogenicity

The virulomes of all *E. coli* displayed high level of pathogenicity (Table 3). The ESBL-*Ec* isolates showed a 93.6% mean probability (P score) of being human pathogens. The pathogenic species with the highest linkage (100% identity) were the *E. coli* APEC O1 (Accession numbers: DQ517526, DQ381420), *E. coli* UMN026 (Accession number: CU928163) and *E. coli* UTI89 (Accession number: CP000243), which are all extraintestinal pathogenic strains in animals (poultry) and humans belonging to the pathogenic phylogroup B2.

The VFs detected belonged to major functional categories including: adhesins, toxins, protectins and invasins, iron uptake/siderophores, anti-phagocytosis, secretion systems and autotransporters (Table 3). The isolate PR85E3 harboured the highest number (72) of VFs, followed by the isolates PN256E2 and PR010E3I with 39 and 24 VFs, respectively. Analysis of the type I fimbriae *fim* showed that it was present in 64% of the isolates. Among putative VFs, autotransporter adhesin *ehaB*, invasin of brain endothelial cells locus B (*ibeB*) and invasin of brain endothelial cells locus C (*ibeC*) belonging to autotransporter protein and invasins, were the most prevalent (73%, 8/11) VFs across the isolates (Table 3 and Table S3). Interestingly, the avian hemolysin gene F (*hlyF*) that enhanced the production of outer membrane vesicles (OMVs) and led to autophagy of eukaryotic cells was detected in one isolate while the hemolysin E (*hlyE*) a pore-forming toxin was observed in 34% (4/11) of isolates.

Our findings showed that rectal *E. coli* isolates harboured significantly more VFs than nasal isolates (141 VFs in rectal isolates vs 104 in nasal isolates, *p* = 0.018). Putative VFs for invasion, such as the outer membrane protein T (OmpT) and the *traT* genes were more prevalent in rectal than nasal isolates. However, the polysialic acid transport protein group 3 (*KpsMIII*) gene encoding for group 3 capsule, was detected only in nasal isolates, as were the unique *vat* and *astA*. Specifically, all *E. coli* isolates harboured at least one ExPEC VF from each of the major functional categories and up to 18 ExPEC VFs (Table 3 and Supplementary Table S1). 

### 2.5. Phylogenetic Groups and Multilocus Sequence Typing, Serotyping and Phylotyping

Based on in silico MLST results, four *E. coli* isolates were assigned to the pandemic ST88 (n = 2) and ST2144 (n = 2) clones, while the remaining isolates were assigned to six single-locus variants, namely, ST10, ST69, ST226, ST944, ST4450 and ST44. Interestingly, the *E. coli* ST2144 were both isolated from two rectal samples processed within the same abattoir (SH004). 

The majority of the isolates were assigned to commensal phylogroups A (45%), B1 (28%) and C (18%) but one belonged to the virulence phylogroup D (9%). The serotype O-:H49 (18.18%) and O-:H18 (18.18%) were the principal serotypes detected, while the *fimH*1250 (18.18%) and *fimH*87 (18.18%) were the predominant *fimH* gene observed. 

### 2.6. Mobile Genetic Elements

WGS analysis identified 15 different plasmid replicons in all the isolates, which further all harboured multiple plasmid replicons concomitantly. Ten types of incompatibility (Inc) plasmid replicons were identified with different frequencies, including IncY, IncFIA, IncFIB (AP001918), IncFIC(FII), IncFII, IncN, IncHI2, IncHI2A, IncI1, IncI2 and IncX (Table 2 and Table 4). The majority of isolates (5/11; 45.45%) harboured the IncF (FII, FIB, FIC, FIA) and IncY (4/11; 36%). Four isolates harbouring the IncF incompatibility group also harboured the IncH (n = 2) and IncI (n = 2) groups. 

In silico plasmid MLST-analyses assigned the IncF plasmid incompatibility group to STs K-:A-:B1 and K89:A-:B57, while IncH and IncI plasmids were assigned to ST3. Additionally, nine (82%) isolates harboured an array of insertion sequences (IS) with IS26, IS421, Isec1 being the most frequent with a 55% prevalence. An array of three IS (IS26, ISVsa3, ISEc9) were harboured on the plasmid IncI that also encoded the sulphonamide resistance gene (*sul2*) and virulence factor (*cib*) in the two *E. coli* ST88 (PR256E1 and PN256E2). Similarly, three isolates harboured transposons (Tn), including Tn6082 (18%) and Tn7 (9%), with the trimethoprim resistance gene *dfrA1* being encoded in transposon Tn7 (Table 4). 

### 2.7. Phylogenetic Analysis

**Figure 1:**
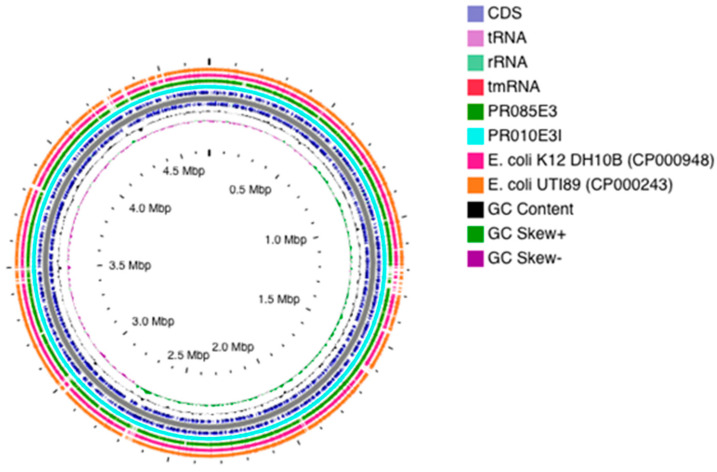
**Circular genome representation of selected ESBL-producing *E. coli* aligned with reference genome and closely related strains.** Circular map of selected ESBL-producing *E. coli* (PR010E3I, PN256E8 and PR085E3) and closely related strains (*E. coli* K12, *E. coli* UTI89, *E. coli* UMN026), with comparative alignment against *E. coli* APEC_O1 (NZ_CP019005.1), generated using CGView Server V1.0. Coloured arrows in the outer ring represent different gene families of the reference genome. A key of the coloured arrows representing different gene families is presented in the inset. The inner coloured circles representing different strains are also listed in the inset. Innermost circles show GC content indicated in black and GC Skew, with green and purple indicating positive and negative values, respectively.

The contigs of the ESBL-*Ec* harbouring the most VFs were mapped against the complete genome of *E. coli* Ecol_AZ155 (NZ_CP019005.1) for visualization of the genomic organisation (Figure 1). The results of the comparative genomic analyses revealed specific similarities and dissimilarities, i.e., isolates had similar and dissimilar arrangements of genomic regions towards representative and reference genomes (Figure 1). 

**Figure 2:**
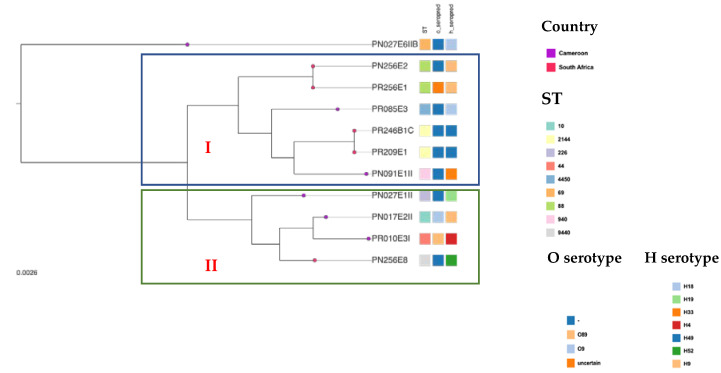
**Comparative genome analysis based on the core genome MLST of study’s ESBL-producing *E. coli* isolates.** Each node represents an isolate, each of which is coloured according to country of origin, as defined in the legend. Clusters of isolates belonging to the same sequence cluster are encircled and annotated. Serotype and sequence types are also indicated via a heatmap. Core-genome phylogenetic tree based on comparison of conserved clusters of orthologous genes (COGs). Interactive map of geographic locations and genetic attributes can be visualized within Microreact at https://microreact.org/project/tYENaUrCix7jMS7RBrFeBi-population-structure-and-pangenome-comparative-analysis-of-esbl-e-coli accessed on 4 June 2022.

Whole genome phylogenetic analysis grouped the study *E. coli* isolates (n = 11) into two major clusters (Figure 2). The first one grouped six isolates including two *E. coli* ST2144 (PR209E1, PR246B1C), two ST88 strains (PR256E1, PN256E2), one ST940 (PN091E1II) and one ST4450 (PR085E3). The two *E. coli* ST2144 isolates identified from the same abattoir (SH004) in South Africa had 100% identity and shared a close common ancestor with *E. coli* ST940 (PN091E1II) and *E. coli* ST4450 (PR085E3), which both originated from one Cameroonian abattoir (SH002). Similarly, the two ST88 isolates (PR256E1 and PN256E2) displayed 100% identity and were found to share common ancestor with the *E. coli* ST2144, ST940 and ST4450. The second clade included four isolates belonging to various STs (i.e., PN256E8: ST9440; PR010E3I: ST44; PN017E1: ST10; PN027E1II: ST226) of which *E. coli* ST10 (PN017E1) and ST44 (PR010E3I) isolated in the same abattoir Cameroon were closely related and shared a common ancestor with *E. coli* ST9440 originating from South Africa.

**Figure 3:**
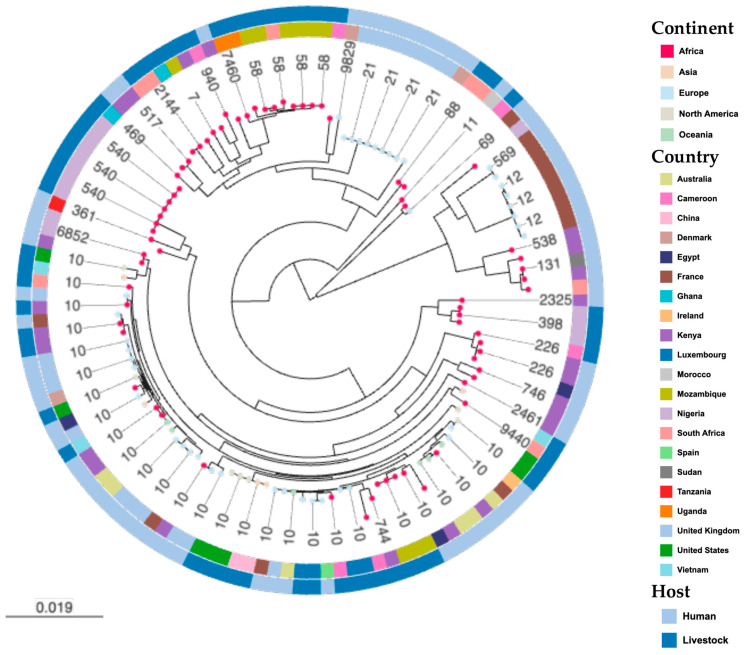
**Phylogenetic relationship between study’s and international *E. coli* isolates.** Isolates of the same continent share the same leaf node colour as depicted in the legend. A total of 118 genomes were used to contextualise our isolates. Mid-point rooted maximum likelihood phylogenetic tree was built using core genome MLST. The inner ring indicates country and the outer ring indicates host source. Interactive map of geographic locations and genetic attributes can be visualized within Microreact at https://microreact.org/project/5CNJcAYrnVvLRLXZX2bXCt-population-structure-and-pangenome-comparative-analysis-of-esbl-e-coli accessed on 4 June 2022.

**Figure 4:**
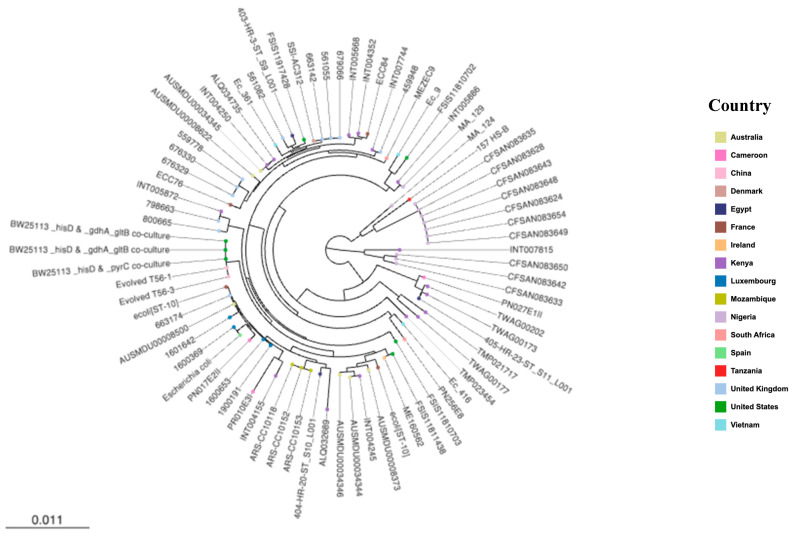
**Phylogenetic relationship between *E. coli* isolates belonging to CC10.** Isolates of the same country share the same colour. Eighty isolates were used to contextualise our isolates. Mid-point rooted maximum likelihood phylogenetic tree was built using core genome MLST.

The phylogenomic analyses of the study’s isolates with international strains revealed that they were more closely related to strains from African countries, such as Kenya, Ghana, Egypt and Morocco, than to any other country (Figure 3). None were phylogenetically related to any strain from the United States or United Kingdom. The genomes from livestock and humans clustered together at some level. Specifically, PR246B1C and PR209E1 (ST2144), described above to have the same virulome, resistome and mobilome, as well as PN091E1II (ST940), were in the same cluster and shared common ancestors with strains isolated from humans and livestock in Kenya, Uganda, South Africa, Mozambique and Ghana (Figure 3). Similarly, hierarchical clustering analyses provided evidence of the genomic relationship between strains originating from livestock and humans with the sole *E. coli* ST10 strain (PN017E2) sharing a common ancestor with the *E. coli* ST10 isolated from humans in Spain and livestock in Luxembourg. Intriguingly, the ST9440 isolate (PN256E8) was closest to an ST10 isolate (Ec416) from livestock in Vietnam (Figure 4). 

## 3. Discussion

In this study, genotypic and pathogenic characteristics of ESBL-*Ec* isolated from pigs collected at Cameroonian and South African abattoirs were investigated using WGS. A diverse population of ESBL-*Ec* harbouring an extensive repertoire of resistance genes and virulence factors have been detected. 

Each genome of ESBL-*Ec* isolated from both countries harboured numerous ARGs, especially the *bla_CTX-M-15_*, supporting other contemporary studies that showed that *bla_CTX-M-15_* is the most prevalent ESBL variant among *E. coli* [13,14]. In fact, Rafai et al., (2015) detected 63.7% of ESBL producers in surgical site infections of humans in the Central African Republic [14]. The authors showed that *bla_CTX-M-15_* was present in all isolates along with *aac(6′)-Ib-cr*. Similar findings were also evidenced by Mbelle et al., (2019) who reported a 70% occurrence of *bla_CTX-M-15_*-carrying strains among hospitalized patients in South Africa [15].

A major observation was the phenotypic multi-drug resistance (MDR) of the isolates PR010E3I and PN256E8, although all isolates, except PR256E1 and PN256E2, harboured concomitant resistance genes encoding for resistance to aminoglycosides, tetracyclines and fluoroquinolones. This finding is in contrast with data readily available from the literature, which suggest that machine or deep learning can be used to predict adequately phenotypic antimicrobial resistance based on genome sequence data [16]. Although transcription analyses could not be undertaken to assess the expression of these genes, these observations led us to posit that the PMQR and QRDR genes, as well as aminoglycoside resistance genes present in these isolates might not have been expressed or be silent. Similar discrepancies regarding the phenomes and genomes of isolates harbouring resistance genes but not expressing associated phenotypic resistance were reported elsewhere [16] and can be further observed in Table 2 and Table S1. Our finding further reveals that the application of machine or deep learning, as well as the comparison between phenome and genome are still needed at a large-scale from various environments and sources. 

All isolates were seen as human pathogens with over 93% of pathogenicity score and the avian pathogenic *E. coli* APEC-O1-ColBM (DQ381420) being the closest related strain. There is increasing evidence that food-producing animals and food products may contribute to the spread of ExPEC in the community [17]. In our study, all ESBL-*Ec* harboured at least three VFs associated with ExPEC, such as *iss*, *iutA*, *traT*, *ompT*, *hlyA*, *iroN*, *papC* and *fimH* [3]. Of great concern is that the isolate PR085EE3 carried over to 72 VFs with the majority being identified in clinical ExPEC. This suggests that commensal bacteria prevailing in the microbiome of food animals are not only reservoirs of resistance genes, but more so, of virulence factors which might be transmitted via horizontal gene transfer to other bacteria and disseminate to humans via the food chain. It reemphasizes the need to ensure adequate food safety measures throughout the farm-to-plate continuum along with effective infection prevention and control measures in hospitals.

The detection of the heat stable enterotoxin 1 (*astA*) gene, encoding for the enteroaggregative *E. coli* heat-stable toxin 1 (EAST1), in the sole ST9440 strain (PN256E8), as well as the avian hemolysin (*hlyF*) in PR256E1 (ST88) gives credence to the fact that commensal *E. coli* prevailing in the gut microbiome have a propensity to acquire various virulence genes, which might, therefore, evolve as progenitor lineages from which heteropathogenic *E. coli,* including uropathogenic *E. coli* (UPEC), neonatal meningitis-associated *E. coli* (NMEC) and enteroaggregative *E. coli* (EAEC) strains, will emerge.

The majority of commensal *E. coli* strains belong to the phylogenetic groups A and B1, whereas the most common virulent ExPEC are associated with group B2 and D. Our ESBL-*Ec* isolates belonged to group A (45%) and B1 (28%). Several reports confirmed that phylogroups A and B1 are the leading phylogroups among *E. coli* isolates especially in the gut microbiome [18,19]. A study from Nigeria showed that 62% of *E. coli* isolates tended towards the commensal phylogroup B1 and A [20]. The relationship between phylogenetic groups and ABR has been established previously [20], and studies have shown that the group B2 strains are mainly MDR [18,21]. However, our study revealed that isolates from other phylogroups, such as group A and B1, could also display MDR and a high level of virulence certainly as a result of horizontal gene transfer. We posit that isolates from other phylogroups, such as group A and B1, though commensals, could also display MDR and high level of virulence, likely due to horizontal acquisition of resistance genes and virulence factors, which might allow commensal bacteria to become putatively virulent in case of extra-intestinal infections. 

The ESBL-*Ec* isolates were mainly circulating in two clonal lineages since four out of seven isolated strains belonged to the ST2144 (n = 2) and ST88 (n = 2). In addition, the MDR-high-risk clone ST69 and the ST10 were also detected. The ST10 complex, including ST10, commonly associated with spread of CTX-M-1, CTX-M-2 and CTX-M-9 groups, is highly distributed among humans and various livestock species and has been linked with intestinal and extra-intestinal infections in several African countries [14]. *E. coli* ST10 was the main ST along with ST131 identified in surgical site infection in the Central African Republic [14]. Like other high-risk clones, *E. coli* ST69 possesses biological factors, such as *usp*, *ompT*, secreted autotransporter toxin (*sat*) and *iutA* genes corresponding specifically to ST131 [13], that increase bacterial fitness allowing these strains to out-compete other bacterial strains and become the principal part of the bacterial population in the gut [13]. The ß-lactamase genes detected in this study is well in line with that described elsewhere in the world. The detection of the *mcr-1* gene in one isolate suggests that colistin-resistant *Enterobacterales* are also emerging among food-producing animals in Africa and demonstrates the urgent need of antimicrobial usage stewardship in food production systems and implementation of effective monitoring programmes to curb the spread of MDR-*E. coli.*

Comparative hierarchical clustering suggested that the majority of our strains belong to two clusters (Figure 2 and Figure 3). Interestingly, in cluster I, the two ST2144 isolates (PR246B1C and PR209E1) originating from South Africa were identical and shared common ancestors with two Cameroonian isolates ST940 (PN091EII) and ST4450 (PR085E3). Likewise, in cluster II, the sole ST9440 isolate (PN256E8) originating from South Africa shared common ancestors with three Cameroonian isolates ST44 (PR010E3I), ST10 (PN017E2I) and ST226 (PN27E1II). This gives credence to the hypothesis that ESBL-*Ec* emerging in one part of the world can spread to another part due to the globalization of trade and international travels [7]. 

The study further confirmed that the ST10 complex is common in African livestock as all our ST10 complex belong to a unique cgMLST cluster containing closely related isolates from Cameroonian (PR010E3I, PN027E1II) and South African pigs (PN256E8). The comparative phylogenomic analysis further confirms that our ESBL-*Ec* ST10 demonstrated overlap with ST10 strains isolated from livestock and human populations in Africa (Mozambique, Egypt, Morocco), Asia (Vietnam), Europe (United Kingdom, Denmark, France) and Oceania (Australia) and display the same phylogroup A. Similarly, our ESBL-*Ec* ST69 share high levels of similarity with an UPEC ST69 phylogroup D that was involved in pyelonephritis in France (unpublished data). This gives credence to the hypothesis that commensal *E. coli* of the gut microbiome might be the reservoir of virulence and resistance genes that allow the emergence of hetero-pathogenic *E. coli* strains [18,19]. 

Mobile genetic elements (MGEs) play an essential role in the mobility of ARGs and VFs between different bacterial species. Our isolates harboured multiple plasmids belonging to major replicon types, especially the IncF (9/11; 81%) plasmid. Similar plasmid replicons associated with *bla_CTX-M-group_* were reported in humans and livestock in Africa and across the world [22]. Given the presence of ESBL-*Ec* in clinically healthy animals and humans, it is likely that the presence of these plasmids could contribute to the long-term persistence of resistance traits in animal and environmental microbiome. 

Though our study was limited by the isolates numbers and geographic area, our results sufficiently reinforce the need to closely monitor pathogenic and commensal bacteria prevailing in the food production systems on the continent [22].

## 4. Conclusions

Our study demonstrates that the population structure of ESBLs-*E. coli* in pigs is highly diverse with the *bla_CTX-M-15_* gene being the leading CTX-M variant. Although the phylogenetic diversity observed precludes any suggestion for clonal dissemination, the resistance and high human pathogenic potential demonstrate the urgent need to implement effective strategies to contain the dissemination of antibiotic-resistant bacteria in Cameroon and South Africa. Our study underlines the necessity of long-term genomic studies investigating commensal and pathogenic bacteria in (food) animals, food products and associated environments, as well as in occupationally exposed workers, in line with the One Health approach, not only to preserve antibiotics for future generations, but also to gain new insights into the diversity, evolutionary history and emergence of ESBL-ExPEC, as basis for sustainable containment of this resistant pathogen.

## 5. Materials and Methods

### 5.1. Study Design and Bacterial Isolates

The study sample consisted of eleven putative ESBL-*Ec* isolates that were collected between March and October 2016 as part of a larger study where ESBL-producing *Enterobacterales* were collected from three abattoirs in Cameroon and two in South Africa. These isolates originating from nasal (n = 6) and rectal swabs (n = 5) from healthy pigs processed at abattoirs, were identified as ESBL producers via VITEK 2 system and as closely related isolates via the enterobacterial-repetitive-polymerase chain reaction (ERIC-PCR) analysis, respectively [12]. They were then selected to assess further the clonal relatedness between Cameroonian and South African isolates.

### 5.2. Identification, ESBL Screening and Antimicrobial Susceptibility Testing

All samples were cultured on MacConkey agar supplemented with 2 mg/L cefotaxime and incubated for 18–24 h at 37 °C in aerobic conditions [7]. All putative ESBL-producers were phenotypically characterized to the genus level using Gram staining and biochemical tests (catalase and oxidase tests). The isolates were thereafter phenotypically confirmed using the VITEK 2 system. 

The VITEK 2 system was further used for ESBL screening along with the double disk synergy test as previously described [12]. A series of 18 antibiotics encompassed in the Vitek^®^ 2 Gram Negative Susceptibility card (AST-N255) were tested using Vitek^®^ 2 System (BioMérieux, Marcy l’Etoile, France). Breakpoints of the CLSI guidelines [8] were used except for the colistin, amoxicillin and clavulanic acid, piperacillin/tazobactam, amikacin for which EUCAST breakpoints [23] were considered with *E. coli* ATCC 25922 and *K. pneumoniae* ATCC 700603 being used as controls. 

### 5.3. Whole Genome Sequencing and Data Analysis

#### 5.3.1. Purification, Sequencing and Pre-Processing of Genomic Data

GenElute® bacterial genomic DNA kit (Sigma-Aldrich, St. Louis, MO, USA) was used for genomic DNA (gDNA) extraction with the concentration and purity assessed using agarose gel electrophoresis, NanoDrop 8000 spectrophotometer (Thermo Scientific, Waltham, MA, USA), and fluorometric analysis Qubit® (Thermo Scientific, Waltham, MA. USA). Libraries were constructed using the Nextera XT DNA Library Preparation kit (Illumina Inc., San Diego, CA, USA) and subjected to paired-end (2×300 bp) sequencing on an Illumina MiSeq (Illumina Inc., San Diego, CA, USA) machine with 100× coverage. The generated paired-end reads were merged, checked for quality, trimmed, and *de novo* assembled into contigs with SPAdes version 3.11 [24]. 

#### 5.3.2. WGS-Based Molecular Typing 

WGS data were used to predict in silico multi-locus sequence type (MLST) based on the Achtman scheme, which considers allelic variation amongst seven housekeeping genes (*adk*, *fum*C, *gyr*B, *icd*, *mdh*, *pur*A and *rec*A) to assign STs [25]. In addition to generating an *E. coli* MLST assignment for each isolate, core-genome MLST (cgMLST) was assigned based on a scheme from EnteroBase server (https://enterobase.warwick.ac.uk/species/index/ecoli accessed on 20 April 2021) that uses 2513 loci [26]. EnteroBase was further used for in silico phylotype predictions following the Clermont scheme [27], as well as for *fimH* allelic designations [26]. Ribosomal MLST, hierarchical cgMLST clustering, wgMLST were further performed using core genome data in EnteroBase. 

#### 5.3.3. In Silico Resistome and Virulome Profiling

ARGs of the *E. coli* genomes were annotated and identified with ResFinder [28] through the bacterial analysis online platform of GoSeqIt tool (www.goseqit.com accessed on 15 November 2020). The Comprehensive Antibiotic Resistance Database (CARD, https://card.mcmaster.ca/ accessed on 20 April 2021) [29] platform was concomitantly used for prediction of ARGs and detection of chromosomal mutations (SNPs) in quinolone-resistant genes of *gyr*A, *gyr*B, *par*C and *par*E. The ARGs predicted are combinations of both database with a threshold set at 90% identity for a positive match between the reference database and a target genome. VirulenceFinder [30] available from the GoSeqIt tools server along with the comparative pathogenomics platform VFanalyzer from Virulence Factor Database (VFDB) [31] were similarly used to predict and annotate virulence factors (VFs), respectively. Likewise, virulence factors detected in our study had a threshold of 90% identity with reference genes and represent combinations of both databases. ExPEC virulence genes, including ferric aerobactin receptor (*iutA*), increased serum survival (*iss*), heat-resistant agglutinin (*hra*)*,* temperature sensitive haemagglutinin (*tsh*), P fimbrial adhesin (*papC*), colicin V (*cvaC*), capsular polysialic acid virulence factor group 2 (*kpsII*) and invasive factor of brain endothelial cells locus A (*ibeA*) of *E. coli* strains responsible for neonatal meningitis in humans were investigated in silico. Moreover, the pathogenicity prediction web-server PathogenFinder [32] was used to predict bacteria pathogenic potential towards human hosts with a threshold ≥90% being considered as isolates with significant pathogenic human potential. 

#### 5.3.4. Detection of Mobile Genetic Elements

The RAST SEED viewer [33] and Artemis Comparison Tool (ACT, [34]) were used to identify the presence of transposases and integrons flanking resistance and virulence genes. MGEfinder was used for the in-silico detection of insertion sequences (IS), conjugative genetic elements and transposons allowing investigation of synteny of mobile genetic elements with VFs and antibiotic resistance genes [35]. PHAge Search Tool Enhanced Release (PHASTER) server was used for the identification, annotation and visualization of prophage sequences [36]. The profile of bacterial plasmid replicons and plasmid incompatibility groups was assessed through PlasmidFinder 2.1 (https://cge.cbs.dtu.dk/services/PlasmidFinder/ accessed on 20 April 2021) and pMLST 2.0 (https://cge.cbs.dtu.dk/services/pMLST/ accessed on 20 April 2021) [37]. Putative CRISP system and Cas cluster were assessed through CRISPRCasFinder (https://crisprcas.i2bc.paris-saclay.fr/CrisprCasFinder/Index accessed on 20 April 2021). 

#### 5.3.5. Genome Visualization and Gene Annotation

The *de novo* assembled raw reads were annotated using the Rapid Prokaryotic Genome (PROKKA) version 1.12 beta available from EnteroBase, the NCBI Prokaryotic Genome Annotation Pipeline (PGAP) and RAST 2.0 server (http://rast.nmpdr.org accessed on 15 November 2020) [38], which identified encoding proteins, rRNA and tRNA, assigned functions to the genes and predicted subsystems represented in the genome. The size, GC content, average coverage, length, N50, L50, RNAs and protein coding sequences were obtained for each isolate. The annotated in silico predicted proteins and regions were visualized via the JSBrowse of EnteroBase server and RAST. The genomes of the isolates were visualized using the CG Viewer Server [39]. In addition, the contigs of selected isolates were mapped against the complete genome of *E. coli Ecol_AZ155* (NZ_CP019005.1) for visualization of the genomic organization.

#### 5.3.6. Comparative Phylogenomic Analyses 

The whole genome phylogenetic relationship was assessed within the study isolates and with a collection of international *E. coli* genomes (*n* = 118) available at the EnteroBase *E. coli* genomes repository as of 12 November 2020 (Supplementary Dataset S1). The international isolates were closely related *E. coli* strains of similar STs isolated from various sources (humans, livestock and environment).

The *E. coli* isolate YA00194039 (ERS4920643) was used as reference genome with all assembled contigs being aligned against it to determine SNP locations. The phylogeny of the *E. coli* isolates was characterised using the whole genome MLST (wgMLST), core genome MLST (cgMLST) and accessory genome MLST. Phylogenetic relationships among study isolates and between study and international isolates were assessed based on nucleotide alignments of all the genes in the entire genome (wgMLST) and core genome content (core genes that are present in most genomes with ≥95% of nucleotide identity; cgMLST). Moreover, the accessory gene, including ARGs, plasmid replicons and phages content, was analysed using the EnteroBase server, which scans the genome against the core ResFinder and PlasmidFinder databases based on a percentage identity of ≥ 90% and coverage of ≥ 70% in order to generate a customized phylogenetic tree to infer the evolutionary relationship within the study isolates and between the study and international isolates. Minimum spanning trees constructed using GrapeTree software and phylogenetic trees were further built to describe the relatedness among the study isolates and between the study and international isolates [40]. The generated phylogenomic trees were downloaded, and, subsequently, visualized and edited using MicroReact (www.microreact.org accessed on 4 June 2022). 

### 5.4. Nucleotide Accession Number

This whole-genome shotgun bioproject **PRJNA548686** of *E. coli* strains PN017E2II, PR010E3I, PN027E6IIB, PR256E1, PN256E2, PN027E1II, PN091E1II, PN256E8, PR209E1, PR246B1C, and PR085E3 has been deposited at DDBJ/EMBL/GenBank under accession numbers VMKK00000000, VKOQ00000000, VKOV00000000, VKOS00000000, VKOT00000000, VKOW00000000, VKOU00000000, QJRZ00000000, VKOO00000000, WHRW00000000, and VKOP000000000 respectively. The versions described in this paper are the versions, VMKK01000000, VKOQ01000000, VKOV01000000, VKOS01000000, VKOT01000000, VKOW01000000, VKOU01000000, QJRZ00000000.1, VKOO01000000, WHRW0000000.1, and VKOP010000000, respectively.

## Figures and Tables

**Table 1:** Genotypic characteristics of ESBL-producing *E. coli* isolates (Bioproject PRJNA412434).

Isolate	Accession Number	Country	Sample Type	Abattoir	MLST *	Clonal Complex	FimH	Phylogroup	Serotype
**PN017E2II**	VMKK00000000	Cameroon	Nasal swab	SH001	10	ST10 Cplx	FimH215	A	O9:H:9
**PR010E3I**	VKOQ00000000	Cameroon	Rectal swab	SH001	44	ST10 Cplx	FimH54	A	O89:H4
**PN027E6IIB**	VKOV00000000	Cameroon	Nasal swab	SH001	69	ST69 Cplx	FimH27	D	O-:H18
**PR256E1**	VKOS00000000	South Africa	Rectal swab	SH005	88	ST23 Cplx	FimH1250	C	O: Uncertain H9
**PN256E2**	VKOT00000000	South Africa	Nasal swab	SH005	88	ST23 Cplx	FimH1250	C	O-:H9
**PN027E1II**	VKOW00000000	Cameroon	Nasal swab	SH001	226	ST226 Cplx	FimH43	A	O-:H19
**PN091E1II**	VKOU00000000	Cameroon	Nasal swab	SH002	940	ST448 Cplx	Unknown	B1	O-:H33
**PN256E8**	QJRZ00000000	South Africa	Nasal swab	SH005	9440	ST10 Cplx	FimH23	A	O-:H52
**PR209E1**	VKOO00000000	South Africa	Rectal swab	SH004	2144	-	FimH87	B1	O-:H49
**PR246B1C**	WHRW00000000	South Africa	Rectal swab	SH004	2144	-	FimH87	B1	O-:H49
**PR085E3**	VKOP000000000	Cameroon	Rectal swab	SH002	4450	-	FimH566	A	O-:H18

* MLST. Multi-locus sequence typing.

**Table 2:** Overview of resistome and mobilome in ESBL-producing *E. coli* isolates.

Isolate	Country	Sample Type	Abattoir	MLST ^#^	β-Lactamase Resistance Genes			Fluoroquinolone Resistance Genes		Other Resistance Genes	Plasmids	pMLST *
					*CTX-M*	*TEM*	*OXA*	QRDR	PMQR			
**PN017E2II**	Cameroon	Nasal swab	SH001	10	*CTX-M-15*	*TEM-1B*	*-*	*-*	*qnrS1*	*aph(6)-Id, aph(3″)-Ib, tet(A), mph(A), sul2, dfrA14,*	IncY, Col(MG828), Col440I, rep21	-
**PR010E3I**	Cameroon	Rectal swab	SH001	44	*CTX-M-15*	*-*	*OXA-1*	*gyrA (p.S83L) gyrA (p.D87N*)	*aac(6′)-Ib-cr*	*aac(3)-IIa, aph(3″)-Ib, aadA5, aph(6)-Id, tet(B), tet(A), sul1, sul2, dfrA17, floR, catB3*	IncFIA, Col440I, IncFII, IncFIB, Col(MG828), rep21	IncF [F36:A20:B1]
**PN027E6IIB**	Cameroon	Nasal swab	SH001	69	*CTX-M-15*	*TEM-1B*	*-*	*-*	*qnrS1*	*strA, strB, sul2, tet(A), dfrA14*	IncY, Col(MG828)	-
**PR256E1**	South Africa	Rectal swab	SH005	88	*CTX-M-1*	*-*	*-*	*-*	*-*	*tet(A), sul2,*	IncI1 ^&^, IncI2, Col(MG828), ColPVC, IncFIB,	IncF [K-:A-:B1]; IncI1[ST3]
**PN256E2**	South Africa	Nasal swab	SH005	88	*CTX-M-1*	*-*	*-*	*-*	*-*	*tet(A), sul2,*	IncI1 **, IncFIB, Col(MG828), Col440I, rep10	IncF [K-:A-:B1]; IncI1[ST3]
**PN027E1II**	Cameroon	Nasal swab	SH001	226	*CTX-M-15*	*TEM-1B*	*-*	*-*	*qnrS1*	*aph(3″)-Ib, aph(6)-Id, tet(A), mdf(A), sul2, dfrA14,*	IncY, Col440I, colRNAI, Col(MG828)	-
**PN091E1II**	Cameroon	Nasal swab	SH002	940	*CTX-M-15*	*TEM-1B*	*-*	*gyrA (p.S83A),*	*-*	*aph(3″)-Ib, aph(6)-Id, aadA1, 16S_rrsC (g.926_926del), tet(B), mph(A), sul2, dfrA1,*	IncX, Col440I	-
**PN256E8**	South Africa	Nasal swab	SH005	944	*CTX-M-55*	*TEM-1B* *TEM-141* *TEM-206*	*-*	*-*	*oqxA, oqxB* *aac(6′)-Ib-cr*	*aac(6′)-Ib3, aadA5, tet(A), sul2, dfrA17, floR, mcr-1.1, fosA3*	IncN, IncHI2A, IncHI2	IncN [ST1]; IncHI2 [ST3-like]
**PR209E1**	South Africa	Rectal swab	SH004	2144	*CTX-M-14*	*-*	*-*	*-*	*oqxB, oqxA*	*aph(3″)-Ib, aph(6)-Id, aadA2b, aadA1, sul3, cmlA1, fosA3*	IncFIC(FII), IncFIB, IncHI2A, IncHI2 rep21	IncF [K89:A-:B57] IncHI2[ST3]
**PR246B1C**	South Africa	Rectal swab	SH004	2144	*CTX-M-14*	*-*	*-*	*-*	*oqxA, oqxB*	*aph(3″)-Ib, aadA2b, aph(6)-Id, aadA1, aph(3″)-Ib, sul3, fosA3, cmlA1*	IncFIC(FII), Col440II, IncHI2A, IncHI2, IncFIB	IncF [K89:A-:B57] IncHI2 [ST3]
**PR085E3**	Cameroon	Rectal swab	SH002	4450	*CTX-M-15*	*-*	*-*	*-*	*qnrS1*	*AadA5, sul2, dfrA17*	IncY	-

^#^ MLST. Multi-locus sequence typing; * pMLST. Plasmid multi-locus sequence typing; QRDR: quinone resistance determining-regions; PMQR: plasmid-mediated quinolone resistance. ^&^ IncI1 (harbours 3 MGEs i.e IS26, ISVsa3, ISEc9 and encoded sul2 and cib); ** IncI1 (harbours 3 MGEs i.e IS26, ISVsa3, ISEc9 and encoded sul2 and cib.

**Table 3:** In silico identification of human pathogenicity and virulence factors in the ESBL-*E. coli* isolates.

Pathogenicity Feature	Nasal Isolates						Rectal Isolates				
	PN017E2II	PN027E6IIB	PN027E1II	PN091E1II	PN256E2	PN256E8	PR010E3I	PR209E1	PR246B1C	PR256E1	PR085E3
**Pathogenicity Score** **(No. of Pathogenic Families)**	0.934 (615)	0.937 (889)	0.94 (526)	0.941 (665)	0.927 (735)	0.932 (625)	0.94 (677)	0.939 (710)	0.937 (682)	0.929 (729)	0.939 (666)
**Human Pathogenicity**	Yes	Yes	Yes	Yes	Yes	Yes	Yes	Yes	Yes	Yes	Yes
**Virulence Factors**											
**Adherence**	*ecp, elf, eae, hcp, fim*	*lpfA,*	*elf, hcp, fim*	*cfa, ecp, elf, eae, hcp, lpfA*	*hra, lpfA, tsh, cfa, ecp, elf, eae, foc, hcp, pap, fim, pil*	*-*	*hra, papA_F19, ecp, elf, eae, hcp, papI, fim,*	*lpfA, cfa, ecp, elf, eae, hcp, papI, fim*	*lpfA*	*pap, foc, lpfA, tsh,*	*hra, lpfA, pap, tsh, cfa, ecp, elf, eae, foc, hcp, fim, lfhA, prl/gapA, cgs, pilW, sta, stf, stgB*
**Autotransporter**	*EhaB*	*-*	*aatA, ehaB, upaG/ehaG*	*ehaB, upaG/ehaG*	*agn43, ehaB, upaG/ehaG,*	*-*	*cah, ehaB*	*eha, upaG/ehaG,*	*-*	*-*	*cah, ehaAB, upaG/ehaG,*
**Iron Uptake**	*-*	*fyuA, irp, sitA,*	*-*	*fyuA, irp, ybt*	*iuc, iut, sitABC, iro, iroN, fyuA, irp, ybt*	*-*	*iut, sitABCD*	*-*	*-*	*irp, iuc, iutA, iroN, fyuA*	*iroN, ccmF, ent, fep, hem*
**Secretion system**	*aec*	*-*	*-*	*aec*	*aec*	*-*	*aec*	*aec*	*-*	*etsC*	*aec, flg, flh, fli, ipaH, gsp, clpB*
**Antiphagocytosis**	*wzc, wzi*	-	-	*wzc, wzi, wbaZ*	-	-	*wzi*	-	-	-	*rmkB, wbjD/wecB, wecC, galF, ugd, wcal, wzc*
**Toxins**	*hlyE*	-	-	-	*hlyF, astA, vat*	*astA*	*hlyE*	*hlyE*	-	*hlyF,*	*hlyAE*
**Protectins and invasins**	*ibeBC*	*KpsE, kpsMIII_K96; iss, ompT*	*ibeBC*	*iss ibeBC*	*KpsE, kpsMIII_K96, iss, ompT, traT, ibeBC*	*ompT,*	*traT, ibeBC, tia*	*iss, ompT, ibeBC,*	*traT, ompT, iss,*	*iss, ompT, traT,*	*ibeBC, che, motA,*
**Miscellaneous**	*espL espX, galE, rmlD,* *gad, terC*	*air, terC, gad, chuA, eilA*	*espL, espX, rmlD*	*espL, espX, terC, gad,*	*mch, mcmA, terC, gad, eilA, air*	*terC, gad,*	*esp, gad, terC, rmlD, galE, cea*	*esp, terC, gad, adeG, air*	*gad, terC*	*cea, cib, mch, mcmA, terC*	*esp, gal, mrsA/glmM, pgi, acpXL, rml, rpoS, phoQ, glnA1, narH, sugC, acrB, farB, icl, mgtB, motB, bioB, katG, gmhA/lpcA, htrB, kdsA, kdtA, lpxABK, msbA, opsX/rfaC, rfa, wecA, air*

**Table 4:** Mobile genetic elements detected in the ESBL-*E. coli* isolates.

Isolate (ST)	Plasmids	Insertion Sequence	Transposons	Phages	CRISPR Array (Cas System)	TR
PN017E2II (10)	IncY, Col(MG828), Col440I, rep21	-	-	-	6 (Cas1)	54
PR010E3I (44)	IncFIA, Col440I, IncFII, IncFIB, Col(MG828), rep21	-	-	-	8 (Cas1, Cas3)	48
PN027E6IIB (69)	IncY, Col(MG828)	ISKpn19, ISEc1, ISEc31, IS4, ISSfl10, IS911, cn_5813_IS911, MITEEc1, ISEc38, IS629, ISEc46, IS5075	-	PHAGE_Entero_mEp460_NC_019716	5 (Cas1)	54
PR256E1 (88)	IncI1 ^&^, IncI2, Col(MG828), ColPVC, IncFIB,	IS26, ISVsa3, ISSbo1, cn_3792_ISSbo1, ISEc9, ISEc40, ISEc38, ISEc13	-	PHAGE_Entero_fiAA91_ss_NC_022750 PHAGE_Shigel_SfII_NC_021857(34) PHAGE_Entero_HK544_NC_019767	6 (Cas2)	51
PN256E2 (88)	IncI1 *, IncFIB, Col(MG828), Col440I, rep10	IS26, ISVsa3, ISEc9	-	-	10 (Cas3)	101
PN027E1II (226)	IncY, Col440I, colRNAI, Col(MG828)	ISKpn19, ISEsa1, IS5075, MITEEc1, IS100, ISEc30, IS5, ISEc26, ISKpn8, IS421, IS609, ISEc38, IS30, IS903	-	-	11 (Cas3, Cas1)	55
PN091E1II (940)	IncX, Col440I	IS6100, MITEEc1, IS421, ISEc30, ISSfl10, IS30, ISEc38, ISEc1, IS100, ISKpn8	Tn7 ^#^	PHAGE_Entero_BP_4795_NC_004813	5 (Cas2)	40
PN256E8 (944)	IncN, IncHI2A, IncHI2	ISVsa3, IS640, IS100, ISEam1, IS30, MITEEc1, ISEc1, ISKpn26, IS421, ISVsa5, IS609	-	PHAGE_Shigel_SfII_NC_021857(34)	8 (Cas2)	87
PR209E1 (2144)	IncFIC(FII), IncFIB, IncHI2A, IncHI2 rep21	IS102, IS629, MITEEc1, ISKpn8, ISVsa5, IS421, IS3, IS26	Tn6082	PHAGE_Shigel_Sf6_NC_005344 PHAGE_Shigel_Sf6_NC_005344 PHAGE_Shigel_SfII_NC_021857(34)	6 (Cas2)	44
PR246B1C (2144)	IncFIC(FII), Col440II, IncHI2A, IncHI2, IncFIB	IS102, IS3, IS629, IS26, ISEc1, ISKpn8, ISVsa5, IS421, MITEEc1	Tn6082	PHAGE_Shigel_Sf6_NC_005344 PHAGE_Shigel_SfII_NC_021857 PHAGE_Entero_fiAA91_ss_NC_022750	8	41
PR085E3 (4450)	IncY	ISVsa3, ISEc9, IS421, ISKpn26, IS3, ISEc1, ISEc38, MITEEc1, IS26, IS102	-	PHAGE_Entero_mEp460_NC_019716 PHAGE_Pseudo_phiPSA1_NC_024365 PHAGE_Entero_fiAA91_ss_NC_022750	4 (Cas3)	39

TR: Tandem Repeat; Synteny of resistance and virulence genes and MGEs; ^&^ IncI1 (harbours 3 MGEs, i.e, IS26, ISVsa3, ISEc9 and encoded *sul2* and *cib*); * IncI1 (harbours 3 MGEs, i.e, IS26, ISVsa3, ISEc9 and encoded *sul2* and *cib*); ^#^ Tn7 (harbouring dfrA1).

## Data Availability

Not applicable.

## Supplementary Materials

The following supporting information can be downloaded at: https://www.mdpi.com/article/10.3390/pathogens11070776/s1, Table S1: Antimicrobial resistance phenotype of the isolates ESBL-*E. coli*; Table S2: Summary of genotypic features of ESBL-*E. coli*; Table S3: Virulence-associated-traits of nasal and rectal ESBL-*E. coli*; Supplementary Dataset S1: Metadata of 129 *E. coli* genomes.
