# Supplementary material for: Genome Analysis of ESBL-Producing Escherichia coli Isolated from Pigs
Source: Pathogens. 2022 Jul 7;11(7):776. doi: 10.3390/pathogens11070776 (PMC9323374; doi:10.3390/pathogens11070776)
Supplement: Supplementary file 1 [file pathogens-11-00776-s001.zip › Supplementary material/Supplementary Table S1.pdf]

Supplementary Table S1. Antimicrobial resistance phenotype of the isolates ESBL-*E. coli*.

Isolate name	β-lactam antibiotics										Non-β-lactam antibiotics						
	AMP	AMC	TZP	CXM	CTX	CAZ	FEP	ETP	MEM	IMP	GEN	AN	CIP	TGC	NIT	CS	SXT
**PN017E2II**	R	S	S	R	R	R	R	S	S	S	S	S	S	S	S	S	R
**PN027E1II**	R	S	S	R	R	R	I	S	S	S	S	S	S	S	S	S	R
**PN027E6IIB**	R	S	S	R	R	R	I	S	S	S	S	S	S	S	S	S	R
**PN091E1II**	R	R	R	R	R	R	R	S	S	S	S	S	S	S	S	S	R
**PR010E3I**	R	S	S	R	R	R	I	S	S	S	R	I	R	S	S	S	R
**PR085E3**	R	S	S	R	R	R	I	S	S	S	S	S	S	S	S	S	R
**PN256E2**	R	S	S	R	I	S	I	S	S	S	S	S	S	S	S	S	R
**PN256E8**	R	S	S	R	R	R	I	S	S	S	R	R	S	S	I	R	R
**PR209E1**	R	S	S	R	I	S	S	S	S	S	S	S	S	S	I	S	R
**PR256E1**	R	S	S	R	R	R	S	S	S	S	S	S	S	S	I	S	R
**PR246B1C**	R	S	S	R	R	S	S	S	S	S	S	S	S	S	S	S	R

AMP: Ampicillin, AMC: Amoxicillin-clavulanic acid; TZP: Piperacillin-tazobactam; CXM: Cefuroxime; CTX: Cefotaxime; CAZ: Ceftazidime; ETP: Ertapenem; MEM: Meropenem; IMP: Imipenem; GEN: Gentamicin; AN: Amikacin; CIP: Ciprofloxacin; TGC: Tigecycline; NIT: Nitrofurantoin; CS: Colistin; TMP/SXT: Trimethoprim-Sulfamethoxazole; S: Susceptible; I: Intermediate; R: Resistant;
