# Supplementary material for: Genome Analysis of ESBL-Producing Escherichia coli Isolated from Pigs
Source: Pathogens. 2022 Jul 7;11(7):776. doi: 10.3390/pathogens11070776 (PMC9323374; doi:10.3390/pathogens11070776)
Supplement: Supplementary file 1 [file pathogens-11-00776-s001.zip › Supplementary material/Supplementary Table S2.pdf]

Supplementary Table S2. Summary of genotypic features of ESBL-*E. coli*

Isolate	GC	N50	L50	No. Contigs	rMLST	wgMLST	Coverage	Length	Genes	RNA	tRNA	CDS total	CDS Coding
**PN017E2II**	50.9	72955	20	270	2011	120277	126	4 614 573	4 626	111	81	4 515	4 354
**PR010E3I**	50.8	69611	21	226	15358	128817	157	4 813 420	4 977	119	80	4 858	4 635
**PN027E6IIB**	50.7	117004	15	179	2135	120284	169	4 970 490	4 946	111	81	4 835	4 696
**PR256E1**	50.5	87060	20	250	14767	128814	131	5 312 214	5 474	105	84	5 369	5 146
**PN256E2**	50.6	101064	16	253	14767	128816	188	5 240 610	5 426	114	89	5 312	5 091
**PN027E1II**	50.9	57128	27	257	1930	128813	116	4 586 694	4 693	111	80	4 582	4 350
**PN091E1II**	50.8	49629	27	250	41587	115069	131	4 822 597	4 871	121	82	4 750	4 535
**PN256E8**	50.6	85550	18	256	32411	128815	128	4 949 938	5 315	108	82	5 207	5 000
**PR209E1**	50.6	76165	22	219	139138	114166	134	5 044 693	5 090	121	84	4 969	4 851
**PR246B1C**	50.5	113392	13	189	139138	11382	171	5 071 043	4 869	108	75	4 761	4 645
**PR085E3**	50.8	57886	28	213	38604	114162	111	4 771 008	4 793	110	79	4 683	4 542

GC: Guanine-Cytosine content, rMLST: ribosomal Multilocus Sequence Type, wgMLST: Whole genome Multilocus Sequence Type, RNA: Ribonucleic acid, tRNA:transfer RNA, Coding sequence
