# Supplementary material for: Genome Analysis of ESBL-Producing Escherichia coli Isolated from Pigs
Source: Pathogens. 2022 Jul 7;11(7):776. doi: 10.3390/pathogens11070776 (PMC9323374; doi:10.3390/pathogens11070776)
Supplement: Supplementary file 1 [file pathogens-11-00776-s001.zip › Supplementary material/Supplementary Table S3.pdf]

Supplementary Table S3. Virulence-associated-traits of nasal and rectal ESBL-*E. coli*

Functional categories traits	Virulence factors	Overall, n=11 (%)	Nasal Isolates (n=5)	Rectal Isolates (n=6)
	**Adhesins**			
papACEFG	P fimbriae	4 (36)	1	3
fimH	Type 1 fimbriae	7 (64)	3	4
hra	Heat-resistant agglutinin	1 (9)	0	1
tsh	Temperature sensitive hemagglutinin	2 (18)	1	1
sfa/foc	S or F1C fimbriae	1 (9)	0	1
	**Toxins**			
hlyD	α-hemolysin	1 (9)	0	1
hlyE	hemolysin E	4 (36)	1	3
hlyA	Hemolysin A	1 (9)	0	1
hlyF	putative avian hemolysin	2 (18)	1	1
astA	Enteroaggregative *E. coli* toxin	1 (9)	1	0
vat	Vacuolating toxin	1 (9)	1	0
	**Siderophore**			
iroN	Salmochelin receptor	2 (18)	1	1
fyuA	Yersiniabactin receptor	3 (27)	3	0
iutA	Aerobactin receptor	2 (18)	1	1
sitA	Periplasmic iron binding protein	3 (27)	2	1
	**Protectins and Invasins**			
kpsM_III	Group 3 capsule	2 (18)	2	0
kpsE	Group 2 capsule	2 (18)	2	0
iss	Increased serum survival	6 (55)	3	3
traT	Conjugal transfer surface exclusion protein	2 (18)	0	2
ompT	Outer membrane protease T	6 (55)	2	4
ibeB	Invasin of brain endothelial cells, IbeA	8 (73)	4	4
ibeC	Invasin of brain endothelial cells, IbeC	8 (73)	4	4
upaG/ehaG	Autotransporter adhesin UpaG and EhaG	6 (55)	3	3
	Autotransporter proteins			
ehaB	Autotransporter adhesin	8 (73)	4	4
